# The Smithsonian Institution

**Published:** 1861-02

**Authors:** 


*The Smithsonian Institution.*—The total amount of the bequest, as received into the treasury, was $515,169 ; interest on the same to July 1846, devoted to the erection of the building, $242,129. In addition to these $135,000 of unexpended income has been invested in State Bonds, so that the present income of the Institution is $38,325,14. The principal expenditures are, for salaries, about $9,000 ; for publications of all kinds, $9,000 ; for meteorological observations, $2,500; for lecturers, $1,000 ; for the library, $3,500 ; for museum, $2,000. There are some incidental matters involving expenditure, and about $5,000 is set apart from the income to make a certain financial change for the sake of economy. The collections of various kinds which had accumulated at Washington, have now been concentrated at the Institution, Congress agreeing to make an appropriation of $4,000 annually to keep them up. They are such as the collection of the Exploring Expedition, under Captain Wilkes, in South America and the South Seas ; that of Lieutenant Herndon's exploration of the Amazon ; Capt. Stanbury's exploration of the Great Salt Lake ; Captain Perry's Japan collections, etc., etc. This museum is stated to be now superior to any other in this country as a general collection, though in the specialties of exotic birds, shells, fossils, and minerals, it is said to be surpassed by the Philadelphia Academy of Natural Sciences. We are glad to see that the Secretary who has charge of this department looks forward to the object of "having a public museum, illustrating as fully as possible the natural history of the world, and taking rank with those of London Paris, Berlin, and Vienna."—*Washington Star.*

